# Genetic and environmental influence on lung function impairment in Swedish twins

**DOI:** 10.1186/1465-9921-11-92

**Published:** 2010-07-06

**Authors:** Jenny Hallberg, Anastasia Iliadou, Martin Anderson, Maria Gerhardsson de Verdier, Ulf Nihlén, Magnus Dahlbäck, Nancy L Pedersen, Tim Higenbottam, Magnus Svartengren

**Affiliations:** 1 Department of Public Health Sciences, Karolinska Institutet, Stockholm, Sweden; 2 Centre for Allergy Research, Karolinska Institutet, Stockholm, Sweden; 3 Department of Pediatrics, Sachs' Children's Hospital, Stockholm, Sweden; 4 Department of Medical Epidemiology and Biostatistics, Karolinska Institutet, Stockholm, Sweden; 5 Department of Clinical Physiology, Södersjukhuset, Stockholm, Sweden; 6 AstraZeneca R&D, Lund, Sweden; 7 Department of Respiratory Medicine and Allergology, Lund University, Sweden; 8 AstraZeneca R&D, Charnwood, UK; 9 Current address: Chiesi Farmaceutici S.p.A., Parma, Italy

## Abstract

**Background:**

The understanding of the influence of smoking and sex on lung function and symptoms is important for understanding diseases such as COPD. The influence of both genes and environment on lung function, smoking behaviour and the presence of respiratory symptoms has previously been demonstrated for each of these separately. Hence, smoking can influence lung function by co-varying not only as an environmental factor, but also by shared genetic pathways. Therefore, the objective was to evaluate heritability for different aspects of lung function, and to investigate how the estimates are affected by adjustments for smoking and respiratory symptoms.

**Methods:**

The current study is based on a selected sample of adult twins from the Swedish Twin Registry. Pairs were selected based on background data on smoking and respiratory symptoms collected by telephone interview. Lung function was measured as FEV_1_, VC and DLco. Pack years were quantified, and quantitative genetic analysis was performed on lung function data adjusting stepwise for sex, pack years and respiratory symptoms.

**Results:**

Fully adjusted heritability for VC was 59% and did not differ by sex, with smoking and symptoms explaining only a small part of the total variance. Heritabilities for FEV_1 _and DLco were sex specific. Fully adjusted estimates were10 and 15% in men and 46% and 39% in women, respectively. Adjustment for smoking and respiratory symptoms altered the estimates differently in men and women. For FEV_1 _and DLco, the variance explained by smoking and symptoms was larger in men. Further, smoking and symptoms explained genetic variance in women, but was primarily associated with shared environmental effects in men.

**Conclusion:**

Differences between men and women were found in how smoking and symptoms influence the variation in lung function. Pulmonary gas transfer variation related to the menstrual cycle has been shown before, and the findings regarding DLco in the present study indicates gender specific environmental susceptibility not shown before. As a consequence the results suggest that patients with lung diseases such as COPD could benefit from interventions that are sex specific.

## Introduction

The adult individuals' lung function is determined both by the maximal level of lung function growth achieved during childhood and adolescence, and by the rate of decline that follows from the early twenties onwards. Both these are likely to be of importance for later development of respiratory disease, such as COPD. Furthermore, factors as FEV_1_, and VC are powerful predictors of mortality [1,2].

In healthy populations, level of lung function is strongly genetically determined both early and later in life, for both men and women [3-6]. Lung function will also be affected by, or co-vary, with other factors, such as smoking and chronic respiratory diseases. However, the relationships between these variables are not always obvious as some smokers never develop symptoms and lung function decline, while some never smokers become ill, etc [1,2]. Interestingly, as smoking behaviour in itself is determined both by genes and environment, it can influence lung function by co-varying not only as an environmental factor, but also by shared genetic pathways [3,7]. Further, both respiratory symptoms and cigarette smoking have been shown to have a sex related co-variance with pulmonary function measures [8,9]. This has brought to attention the possibility that an individual's genes affect his or her sensitivity to factors important for respiratory health [3].

Therefore, the objective of the current cross-sectional study in a Swedish sample of twins was to evaluate heritability for different measures of lung function, and to investigate, by sex, how the estimates are affected by the covariates smoking and respiratory symptoms.

## Materials and methods

### Study population

The current study (approved by the Ethical Committee at Karolinska Institute, # 03-461) is based on a selected sample of twins born 1926-1958 from the population based Swedish Twin Registry [10,11] who were contacted using a computer-assisted telephone interview in 1998-2002. The interview included a checklist of common diseases and respiratory symptoms, as well as smoking habits [10,11]. Details are shown in the online appendix. From the population of 26,516 twins in pairs where both participated in the telephone interview, 1,030 twins in 515 pairs were selected to participate in more in-depth measures of lung function. The subjects gave written informed consent to participate in the study. To assure that the sample would contain twins with symptoms of respiratory disease (self-reported symptoms of cough, chronic bronchitis, emphysema or asthma) disease concordant and discordant twins were prioritized over symptom free twin pairs. Due to the relatively small number of symptom concordant twins available in the population, pairs were included regardless of smoking habits, while symptom discordant and symptom free pairs were further stratified according to whether none, one, or both of the twins in a pair had a significant smoking history, i.e. had smoked more than 10 pack years (1 pack year is equal to smoking 20 cigarettes per day for 1 year) at the time of inclusion. Table 1 describes the number of twins with the specified combinations of symptoms/smoking habits available from the Swedish Twin Registry. In order to reach the desired number of twin pairs in each category, it was necessary to invite twins from the whole country, as well as twins over a relatively large age span (from 50 yrs with no upper limit), to the study hospital, situated in Stockholm, Sweden. In total, 392 twins (38%) of 1,030 twins accepted the invitation to participate. Two of the 392 twins participated only by sending in the questionnaire due to poor health. Technically acceptable forced expiratory volume in one second (FEV_1_) and vital capacity (VC) measurements were performed by 378 individuals, resulting in 181 complete pairs. Five individuals had incomplete information on smoking habits, resulting in 176 complete twin pairs available for covariate analysis. The corresponding figures for acceptable single breath carbon monoxide diffusing capacity (DLco) measurements were 375 individuals in 178 complete twin pairs. After excluding those with missing smoking data, 173 complete pairs remained.

**Table 1:** Available, invited and participating twins from the Swedish Twin Registry.

Group	1	2	3	4	5	6	Total
Symptoms: twin1, twin2	++	--	--	--	-+	-+	
Smoking > 10 PY: twin1, twin2		--	-+	++	--	++	

No of available	834	12,008	7,708	4,158	1,050	758	26,516
No of invited	394	128	164	106	86	152	1,030
No of participating	130	56	79	43	42	42	392
Participation in % of available	15.6	0.5	1.0	1.0	4.0	5.5	1.5

Groups: 1) Both have respiratory or minor respiratory symptoms, 2) Both healthy, neither have > 10 pack years, 3) Both healthy, one twin with > 10 pack years, 4) Both healthy, both have > 10 pack years, 5) One twin with respiratory symptoms, one healthy, neither have > 10 pack years, 6) One twin with respiratory symptoms, one healthy, both have > 10 pack years,.

### Lung function testing

All lung function tests were carried out in a single specialized clinic with highly experienced staff. Lung function in terms of FEV_1_, VC and DLco was measured according to American Thoracic Society criteria [12,13], using a Sensormedics 6200 body plethysmograph (SensorMedics; Yorba Linda, CA, USA). Each subject performed several slow and forced vital capacity expirations. FEV_1 _was compared to the largest obtained VC and individuals with an obstructive pattern (an FEV_1_/VC ratio 5 units below the predicted value, or FEV_1 _below 90% of the predicted value) also performed a new test 15 minutes after bronchodilation with a short-acting beta2-agonist (nebulized Salbutamol). The maximum values for VC and FEV_1 _(measured pre- or post bronchodilation) were then used for analysis. Based on lung function, twins could be classified according to GOLD-criteria [14].

Self reported cigarette smoking was assessed at the clinical examination and quantified as pack years.

### Determination of twin zygosity

Zygosity of the sex-liked pairs was determined by the use of a set of DNA markers from blood drawn at the clinical testing. Blood samples were not available for both members in 14 pairs, and zygosity information for these twins was instead obtained at the time of registry compilation on the basis of questions about childhood resemblance. Four separate validation studies using serology and/or genotyping have shown that with these questions 95-98% of twin pairs are classified correctly [11].

### Statistical methods

Respiratory symptoms and pack years were assessed as covariates in the linear multivariate regression models stratified by sex. Analyses were performed with the Stata 9.2 software package (StataCorp LP, College Station, TX, USA)

### Quantitative genetic analysis

Quantitative genetic analysis aims to provide estimates of the importance of genes and environment for the variation of a trait or disease (phenotype). The phenotypic variance is assumed to be due to three latent, or unmeasured, factors: additive genetic factors (a^2^), shared environmental factors (c^2^) *or *dominant genetic factors (d^2^), and non-shared environmental factors (e^2^), which also include measurement error. Heritability is a term that describes the proportion of total phenotypic variation directly attributable to genetic effects [15]. Twins are ideal for these types of studies as we know how they are genetically related: identical (monozygotic (MZ)) twins share the same genes, whereas fraternal (dizygotic (DZ)) twins share, on average, half of their segregating genes. We also assume that shared environment (for example the presence of a childhood cat, or parental socioeconomic status) contributes to within-pair likeness to the same extent in MZ and DZ twin pairs. By calculating similarity within and between MZ and DZ twin pairs, we can obtain information about the importance of genetic and environmental factors to the variance of the trait in question. One such measure of twin similarity is the intra-class correlation (ICC) [16].

**Figure 1:**
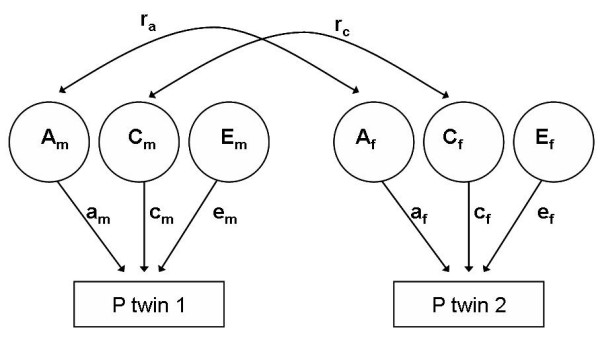
**Basic path diagram for an opposite sexed twin pair**. A_m_, C_m_, E_m_, A_f_, C_f_, and E_f _are the genetic, shared and non-shared environmental variance components for men and women, respectively. The genetic correlation, r_a_, is set free to be estimated in the model, while the shared environmental correlation, r_c_, is set to 1.

These assumptions can also be illustrated in a path diagram, representing a mathematical model of how genes and environment are expected to contribute to phenotypic variance [11]. Figure 1 illustrates a path diagram for an opposite-sex twin pair. The additive genetic correlation (ra) is set to 1 in MZ twins and 0.5 in like-sex DZ twins, based on how genetically related they are, as described above. The shared environment correlation (r_c_) is the same for MZ and DZ twins and therefore set to 1 for both groups. By definition there is no correlation for the non-shared environment. The additive genetic, shared, and non-shared environmental variance components are noted as a_m_, c_m_, e_m_, a_f_, c_f_, and e_f_, for men and women, respectively. The dominant genetic correlation, not included in this figure, is set to 1 in MZ twins and 0.25 in like-sex DZ twins. The simultaneous estimation of c^2 ^and d^2 ^is not possible because of statistical issues [17]. However, which factor should be modelled is suggested by the ICC, where d^2 ^is only included in the model if the correlations of DZ twins are less than half the correlations of MZ twins.

In order to test for sex differences, i.e. whether the same genes and environment contribute to the phenotypic variance in both men and women, different versions of the models can be compared [18]. In the first variance model, we allow the genetic and environmental variance components to be different for men and women, and the genetic correlation (r_a_) is free to be estimated for opposite-sexed DZ twins. For instance, if the genetic correlation is estimated at 0, it indicates that completely different genes influence the trait in men and women. Variance model 2 tests whether the genetic and environmental variance components are allowed to be different for women and men (e.g. if genetic variance is more important in men than in women), constraining the genetic correlation for members of the opposite-sex twin pairs to 0.5. Variance model 3 has equal genetic and environmental variance components for men and women. If the fit of this models is not significantly different compared to the previous, we can assume that there are no sex differences in the magnitude of genetic and environmental influences. In summary, the difference in chi-squares between nested models is calculated in order to test which of the models fits better. A significant chi square difference indicates that the model with fewer parameters to be estimated fits the data worse. Model fitting was performed with the Mx program [19].

All models were tested using lung function in percent of predicted value, the same adjusted for pack years, and finally also adjusted for presence of respiratory symptoms.

## Results

### Descriptive statistics

A summary of the available and participating twins is presented in table 1.

Lung function results and covariates (age, height, pack-years and symptoms) are presented by zygosity group in table 2 and were found to influence independently and significantly each lung function measure (p < 0.05). GOLD stages for twins with lung function data were: Stage 1 - 53 twins (15% of the cohort), stage 2 - 42 twins (12%), stage 3 and above - 2 twins (1%).

**Table 2:** Mean value (± Standard Deviation) for lung function measures and covariates, by sex and zygosity.

	Men		Women		Opposite sexed pairs (n = 42)	
			
	MZ (n = 28)	DZ(n = 14)	MZ(n = 65)	DZ(n = 27)	Men	Women
Age	60.5 ± 9.0	59.8 ± 7.3	59.1 ± 8.3	60.1 ± 9.6	58.5 ± 8.7	58.5 ± 8.8
Height	178.7 ± 5.7	180.1 ± 5.3	164.4 ± 6.2	163.9 ± 5.5	178.3 ± 5.5	165.0 ± 4.8
Pack yrs^1^	11.6 ± 17.6	22.4 ± 20.2	9.5 ± 14.0	13.6 ± 16.9	15.5 ± 16.2	11.9 ± 16.5
VC in % pred.	100.64 ± 13.00	97.86 ± 15.83	111.99 ± 15.42	108.89 ± 13.94	101.97 ± 13.04	113.81 ± 14.90
FEV_1 _in % pred.	92.97 ± 14.91	89.70 ± 18.37	98.96 ± 16.60	98.21 ± 15.77	96.27 ± 15.53	102.68 ± 14.99
DLco^2 ^in % pred.	95.06 ± 18.44	92.45 ± 19.58	87.11 ± 16.08	79.97 ± 14.54	91.83 ± 18.99	89.33 ± 15.97

Multiple regression was used in order to test for differences in the mean levels of age, height, packyears and lung function measures (VC, FEV_1 _and DLco) between the zygosity groups. Adjustment for sex was made for height and lung function measures. ^1^Packyears at examination. ^2 ^n pairs MZ male = 28, DZ male = 15, MZ female = 66, DZ female = 25, OS = 39.

### Intraclass correlations

Intraclass correlations (ICC) for unadjusted and adjusted lung function variables are presented in table 3. Comparing ICC for MZ and DZ twins, the presence of additive genetic influences (ICC for MZ twins > 2 × ICC for DZ twins) was indicated for all measures, except for FEV_1 _in men, where DZ twins showed similar or higher correlation compared to MZ twins, indicating that additive genetic influences are of less importance. For DLco in women, MZ correlations were more than twice as high as DZ correlations, showing evidence of genetic dominance. Sex differences were also indicated for FEV_1_, as unlike sexed DZ twins had lower ICC compared to same-sex DZ.

**Table 3:** Intraclass correlations (with 95% confidence intervals) for unadjusted and adjusted FEV_1_, VC and DLco in a Swedish twin sample by sex and zygosity status.

	**Men**		**Women**		**OS**
			
	**MZ (n = 28)**	**DZ (n = 14)**	**MZ (n = 65)**	**DZ (n = 27)**	**(n = 42)**
			
**VC**					
			
Unadjusted	0.57(0.26;0.78)	0.41(-0.16;0.77)	0.67(0.50;0.78)	0.18(-0.21;0.53)	0.23(-0.08;0.50)
			
Adj. PY	0.49(0.15;0.73)	0.29(-0.29;0.71)	0.67(0.50;0.78)	0.28(-0.12;0.59)	0.25(-0.06;0.52)
			
Adj. PY, symptoms	0.36(-0.01;0.65)	0.18(-0.39;0.65)	0.66(0.50;0.78)	0.26(-0.14;0.58)	0.24(-0.07;0.51)
			
**FEV_1_**					
			
Unadjusted	0.28(-0.10;0.59)	0.42(-0.14;0.78)	0.67(0.50;0.78)	0.32(-0.07;0.62)	-0.07(-0.37;0.24)
Adj. PY	0.27(-0.12;0.58)	0.25(-0.33;0.69)	0.66(0.50;0.78)	0.39(0.01;0.67)	-0.01(-0.31;0.30)
Adj. PY, symptoms	0.18(0.21-0.52)	0.18(-0.39;0.65)	0.65(0.48;0.77)	0.32(-0.06;0.63)	-0.09(-0.38;0.22)
**DLCO**					
Unadjusted	0.58(0.27;0.79)	0.65(0.21;0.87)	0.46(0.24;0.63)	0.06(-0.35;0.44)	0.35(0.04;0.60)
Adj. PY	0.37(-0.00;0.65)	0.23(-0.32;0.67)	0.41(0.19;0.60)	0.01(-0.39;0.40)	0.29(-0.03;0.56)
Adj. PY, symptoms	0.38(0.00;0.66)	0.29(-0.26;0.70)	0.41(0.17;0.58)	0.02(-0.38;0.41)	0.29(-0.03;0.56)

^1 ^n pairs MZ male = 28, DZ male = 15, MZ female = 66, DZ female = 25, OS = 39. PY= pack years

### Sex differences in the genetic influence on measures of lung function

In order to test for sex differences, structural equation variance models with different assumptions regarding the influence of genetic and environmental effects in men and women were fitted based on the ICC results. The models were then compared to each other to find the most parsimonious one fitting our data. Specific variance model fitting results are available in the online appendix (table 4).

**Table 4:** Fit statistics from structural equation modelling for VC.

	**-2LL**	**df**	**AIC**	**Diff Chi-2**	**Diff df**	**p**
	
**Unadjusted**						
Model 1	2827,767	343	2141,767			
Model 2 vs. 1	2827,870	344	2139,870	0,103	1	0,748
Model 3 vs.2	2829,011	347	2135,011	1,141	3	0,767
						
**Adj. PY**						
Model 1	2812,858	341	2130,858			
Model 2 vs.1	2812,859	342	2128,859	0,001	1	0,976
Model 3 vs.2	2815,019	345	2125,019	2,161	3	0,540
						
**Adj. PY, sympt**.						
Model 1	2805,169	339	2127,169			
Model 2 vs.1	2805,215	340	2125,215	0,046	1	0,830
Model 3 vs.2	2809,601	343	2123,601	4,386	3	0,223

Models: 1) rg (genetic correlation) free for DZ opposite-sex twins, ACE different for men and women. 3) rg fixed at 0.5 for DZ OS, ACE different for men and women. 4) rg fixed at 0.5 for DZ OS, ACE same for men and women, df = degrees of freedom, PY= pack years

**Figure 2:**
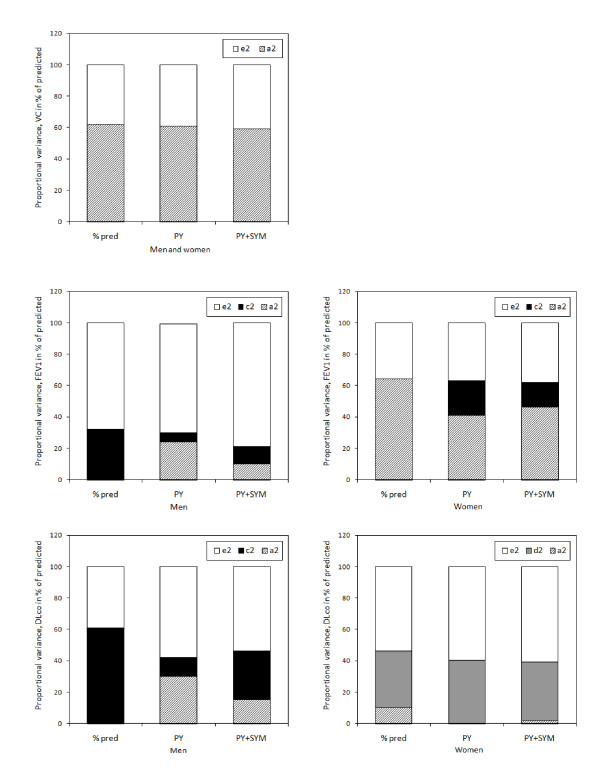
**Unadjusted and adjusted genetic, shared and non-shared environmental variance components for VC, FEV_1_, and DLco in men and women**. Variance is expressed in absolute numbers and is shown for unadjusted data (% pred), data adjusted for pack years (PY) and data adjusted for pack years and respiratory symptoms (PY + SYM).

In summary, a model including additive genetic factors (A), shared environmental factors (C) and non-shared environmental factors (E) was used for VC and FEV_1_. The comparisons of variance models indicated that the importance of genetic and environmental effects was the same in men and women (figure 2). For FEV_1_, the same genes are of importance for men and women (comparing model 2 and 1 in table 5), but the influence of genes and environment differs by sex (significantly different fit between model 2 and 3).

**Table 5:** Fit statistics from structural equation modelling for FEV_1_.

	**-2LL**	**df**	**AIC**	**Diff Chi-2**	**Diff df**	**p**
	
**Unadjusted**						
Model 1	2908,199	343	2222,199			
Model 2 vs. 1	2908,199	344	2220,199	0,000	1	0,986
Model 3 vs. 2	2916,168	347	2222,168	7,969	3	0,047
						
**Adj. PY**						
Model 1	2864,305	341	2182,305			
Model 2 vs. 1	2864,305	342	2180,305	0,001	1	0,980
Model 3 vs. 2	2171,283	345	2181,283	6,978	3	0,073
						
**Adj. PY, sympt.**						
Model 1	2845,428	339	2167,428			
Model 2 vs. 1	2845,448	340	2165,448	0,021	1	0,885
Model 3 vs. 2	2853,815	343	2167,815	8,367	3	0,039

Models: 1) rg (genetic correlation) free for DZ opposite-sex twins, ACE different for men and women. 3) rg fixed at 0.5 for DZ OS, ACE different for men and women. 4) rg fixed at 0.5 for DZ OS, ACE same for men and women df = degrees of freedom, PY= pack years

For DLco, separate models had to be fitted from the start for men and women. For men, a model containing A, C and E was used (as above), while a model including A, E and dominant genetic factors (D) was used for women, since there was evidence for genetic dominance in the latter group (table 6).

**Table 6:** Fit statistics from structural equation modelling for DLco.

Men ACE	-2LL	df	AIC
	
DLco: unadjusted	727,511	82	563,511
DLco: adj. PY	708,851	81	546,851
DLco: adj. PY, symptoms	703,134	80	543,134
**Women ADE**	-2LL	df	AIC
	
DLco: unadjusted	1507,060	178	1151,060
DLco: adj. PY	1491,184	177	1137,184
DLco: adj. PY, symptoms	1490,454	176	1138,454

PY= pack years

### Contribution of genes and environment to the total variance

**Figure 3:**
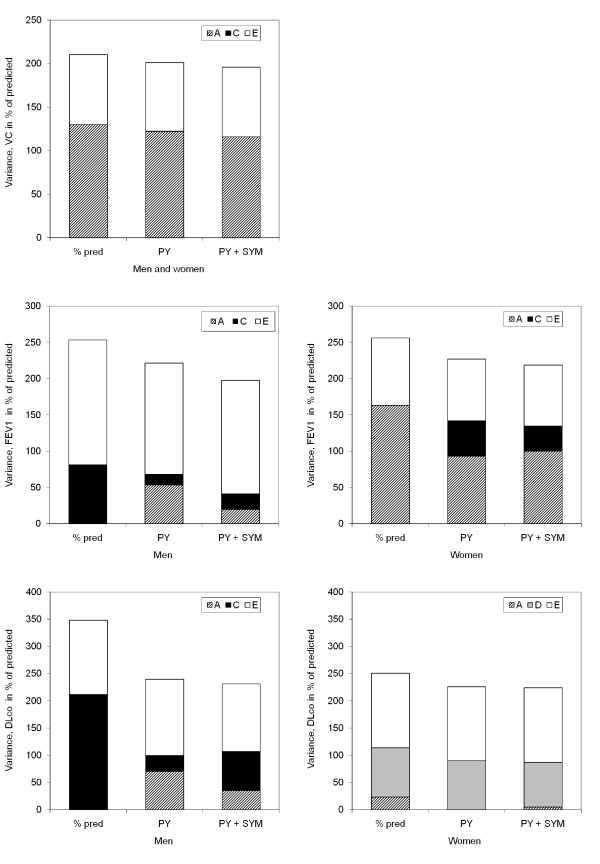
**Unadjusted and adjusted genetic, shared and non-shared environmental variance components for VC, FEV_1_, and DLco in men and women**. Variance is expressed as percentage of total variance and is shown for unadjusted data (% pred), data adjusted for pack years (PY) and data adjusted for pack years and respiratory symptoms (PY + SYM).

Figure 3 shows the variance in absolute numbers (A + C + E = absolute total variance), while figure 2 shows the extent to which genetic and environmental factors contributed to the total variance (%a^2 ^+ %c^2 ^+ %e^2 ^= 100% of total variance).

Unadjusted data show that mainly genetic, but also non-shared environmental influences were of importance for the variance of VC. For FEV_1 _and DLco, analyses had to be separated by sex, as indicated above. For both measures, the variance was attributable to both genetic and environmental factors for women, but only to environmental factors in men (figure 2).

### Influence of smoking and symptoms on the total variance

Adjustment for pack-years and respiratory symptoms resulted in a decrease of the total variance of all lung function measures (VC, FEV_1_, and DLco) between 7 and 37%.

For VC, the decrease in total variance was due to a small reduction of genetic variance, whilst the non-shared environmental variance was stable after adjustments. For FEV_1 _and DLco, the effect of smoking and symptoms was found to be larger in men than in women. The total variance decrease was due to a reduction attributed to genetic variance in women, and shared environmental variance in men.

## Discussion

In the current study all lung function measures (VC, FEV_1 _and DLco) were shown to be influenced by genetic factors. FEV_1 _and DLco showed sex differences in the relative importance of genes and environment, as well as in how smoking and respiratory symptoms influence the genetic and environmental estimates of the trait. Heritability of FEV_1 _has been studied before, but for the gas transfer measure DLco, related to clinical findings such as emphysema, the information is new. VC heritability was higher, and without sex differences.

The relationship between smoking and the presence of respiratory symptoms as well as impaired lung function has been long known. More recently, studies of general twin populations have suggested that genetic factors are of importance in individual differences in lung function [4,6,20], and family studies have shown that relatives of subjects with COPD had a higher risk of airflow obstruction than controls [21-23]. In another study of unselected elderly twins in the Swedish twin registry [6], heritability estimates adjusted for smoking were lower for FEV_1 _(24-41% vs. 67%) but more similar for VC (61% vs. 48%) than the current results. In that study no sex differences were found in heritability estimates, but opposite sexed pairs were not included, reducing power to find such differences. Heritability is population specific and will differ between samples that differ in the distribution of environmental risk factors. Even though both populations were of the same nationality and age range, the prevalence of smoking habits and symptoms would have been lower in the unselected second material, which could explain why differences between studies were seen particularly for FEV_1_, which, as stated above, is known to be susceptible to these factors.

Genes and environments contributed differently to the lung function parameters for men and women. Since it is known that genes influence smoking habits, we chose to present results both with and without the adjustment of pack years. Symptoms on the other hand can be a part of COPD. Adjusting for symptoms give estimates of heritability for lung function not associated with symptoms. The differing results for how much these covariates influenced the three measures used in the current study are not unreasonable as the measures represent different aspects of lung function. Smoking induced pathology is likely to be primarily seen in FEV_1 _and DLco, while VC can remain, at least initially, essentially normal. The approach of adjusting for covariates can however underestimate heritability for the disease itself particularly if they share genetic and environmental effects in common. In women, smoking accounted for some part of the genetic variance, while in men, it accounted for parts of the shared environmental variance. It has been suggested that due to differences in how smoking has been socially accepted in men and women, it is possible that men are more influenced by cultural reasons for smoking than women [24]. Another explanation to the apparent greater proportion in genetic variance in women could be that there is a reduction in absolute range of environmental variance for smoking in women during the mid 1900's, resulting in a proportionate greater observed genetic variance. Thus, genetic variance *per se *may not have changed, but when seen in relation to environmental variance, it appears to have increased [24].

Hence, smoking and respiratory symptoms could have differential effects on the variance components of men and women. The literature also provides examples of sex-specific effects on different aspect of lung function by genetic determination of physiological and hormonal patterns, such as the connection between aerobic capacity and risk for development of COPD [25], the impact of oestrogen on smoke toxins in the lung [26], and variations in gas transfer over the menstrual cycle [27].

On the phenotypic level, sex differences in the relationship between smoking and lung function impairment have been described in the literature, where women smoke less but have more severe lung function decline and more symptoms [3,8,9]. Two recent studies [28,29] have suggested the presence gene-environment interaction, i.e. that the effect of smoking on lung function (here FEV_1_) is dependent on the genotype of the individual. Given our results, we suggest that there are sex differences of importance to the genetic and environmental influence on lung disease. However, the number of twin pairs in the current study was insufficient to evaluate possible interactions. Age is an important covariate in lung disease, but in the current study the number of participants prevented further modelling.

## Conclusions

We have in a sample of twins with and without smoking and respiratory symptoms, shown that not only environment but also genes determine the variability in VC, FEV_1 _and DLco. Differences between men and women were found in the size of the relative importance of genes, but also in the type of genetic pattern (additive vs. dominant). Adjustments of smoking and respiratory symptoms had also differential effect on men and women for FEV_1 _and DLco. Further studies on gene-environment interaction are needed to fully understand the sex differences in respiratory disease. The present study highlights the importance of evaluating genetic and environmental influence on lung function by sex, and suggests that patients with lung diseases such as COPD could benefit from interventions that are sex specific both on the genetic and environmental level.

## Appendix

### Specification of questions from the SALT questionnaire

1. Do you have recurrent periods of coughing?

If yes:

2. Do you regularly cough up phlegm?

Do you have or have you had:

3. Chronic bronchitis (bronchitis)

4. Emphysema

Chronic bronchitis was defined as:

Recurrent cough with phlegm production, and/or self reported chronic bronchitis and/or self reported emphysema (positive answers to questions 1+2 or 3 or 4).

## Competing interests

JH, AI, MA, NLP and MS declare that they have no competing interests.

MGdeV, UN and MD are employed at AstraZeneca and own stocks from AstraZeneca. TH was employed at AstraZeneca when the paper was initiated, current affiliation Chiesi Farmaceutici.

## Authors' contributions

JH planned and conducted the study, as well as drafted the manuscript and did most of the statistical analyses. AI did the calculations on heritability. MA assisted JH in spirometry issues and supervised the test-leaders. NLP, in charge of the Swedish Twin Registry contributed with valuable knowledge on twin heritability aspects. MS conceived of the study, and participated in its design and coordination. MGdV, UN, MD, TH, MA and MS all participated in the monthly meetings and contributed to the writing of the final paper. All authors read and approved the final manuscript.
